# Five-year trends in psychiatric inpatient care: insights from 5,887 patients on substance use, involuntary admissions, and treatment approaches

**DOI:** 10.3389/fpsyt.2026.1778865

**Published:** 2026-02-11

**Authors:** Emine Merve Akdag, Aybeniz Civan Kahve, Rukiye Ay Diker, Serap Aydın, Zeynep Arslan Barlas, Tuğba Aktürk

**Affiliations:** 1 Department of Psychiatry, University of Health Sciences, Bursa Yüksek Ihtisas Training and Research Hospital, Bursa, Türkiye; 2 Department of Psychiatry, Gazi University Faculty of Medicine, Ankara, Türkiye; 3 Department of Psychiatry, University of Health Sciences, Ankara Bilkent City Hospital, Bilkent, Ankara, Türkiye

**Keywords:** involuntary hospitalization, pharmacological treatment, psychiatric inpatient, substance use disorder, trend analysis

## Abstract

**Objective:**

This study aimed to examine the prevalence and temporal trends of SUDs among psychiatric inpatients, investigate the factors predicting involuntary hospitalization, and assess the impact of SUDs and involuntary admission on pharmacological treatment practices.

**Methods:**

A retrospective cross-sectional analysis was conducted using medical records of all psychiatric inpatients (n = 5,887) admitted to the general psychiatric wards of a regional training and research hospital between 2019 and 2023. Descriptive statistics, logistic regression, linear regression, and seemingly unrelated regression (SUR) analyses were employed to evaluate clinical predictors and treatment trends.

**Results:**

The prevalence of SUD was 27.5% (n = 1,619), with mixed substance use being the most common pattern (76.1%). In the logistic regression model, substance use, male sex, and later years of admission were associated with a higher likelihood of involuntary hospitalization, whereas bipolar affective disorder was associated with a lower likelihood. Treatment trends showed an overall increase in atypical antipsychotics, depot antipsychotics, clozapine, and electroconvulsive therapy (ECT); compared with non-users, patients with SUD showed lower use of typical antipsychotics, mood stabilizers, clozapine, and ECT.

**Conclusions:**

This large-scale study demonstrates that SUDs are a strong predictor of involuntary psychiatric hospitalization and significantly influence pharmacological treatment patterns. The increasing trend of compulsory admissions, particularly among male patients with SUD, underscores the urgent need for clearer legal and ethical frameworks, as well as the expansion of community-based mental health services in Türkiye.

## 1 Introduction

Illicit substance use is a global public health concern that threatens not only individuals’ mental health but also their physical well-being, imposes a substantial burden on health care systems, and results in serious societal consequences. According to the 2025 World Drug Report, the number of people using psychoactive substances, excluding alcohol and tobacco, has exceeded 316 million (1). Substance use disorders (SUDs), depending on the type of substance used, duration of use, genetic predisposition, and individual factors, lead to impairments in cognitive, social, and emotional development, resulting in loss of functioning and marked deterioration in quality of life (2). Moreover, behavioral and environmental consequences arising from substance use are strongly linked to broader societal challenges, including increased rates of interpersonal violence, criminal justice involvement, and other adverse social outcomes (3). The global burden of SUDs, when assessed in terms of disability-adjusted life years (DALYs), has been found to be particularly high among young individuals and males, especially in high-income countries (4, 5). In recent years, the increasing diversity of psychoactive substances and the expansion of their patterns of use have further contributed to the aggravation of this burden.

The significance of substance use disorders (SUDs) is not limited to the consequences of addiction itself. When co-occurring with other psychiatric disorders, SUDs complicate the clinical presentation, introduce additional risks, and make patient management more challenging. Hoblyn et al. (2009), in a study evaluating 2,963 patients with bipolar disorder, reported that alcohol use disorder and polysubstance dependence were the strongest risk factors for psychiatric rehospitalization; notably, in divorced patients with both alcohol and polysubstance use disorders, the risk of rehospitalization reached 100% (6). Benz et al. (2023) reported that among 1,315 patients diagnosed with major depressive disorder, 38.4% had a comorbid substance use disorder, and within this group, the use of more than three psychotropic medications was common. In particular, patients with comorbid opioid use disorder were found to have markedly increased rates of risky medication combinations and adverse effects (7). Schmidt et al. (2011), in a 15-year follow-up study of patients with schizophrenia, demonstrated that those with comorbid substance use disorders had two to three times more psychiatric hospitalizations and emergency visits, a significantly reduced average life expectancy, substance use listed as a contributing factor in 30% of deaths, and higher rates of involuntary admissions (8). These findings indicate that, when co-occurring with psychiatric conditions such as depressive disorder, bipolar disorder, and schizophrenia, substance use disorders (SUDs) negatively affect not only symptom severity but also hospitalization rates, treatment processes, involuntary admissions, and overall life expectancy. In a nationwide study conducted in Norway including more than 25,000 patients receiving specialized mental health services, substance use was reported to be twice as common among men, with the highest prevalence observed in male inpatients aged 18–29 years. The study further revealed that alcohol and sedative use were particularly prominent among inpatients with anxiety disorders, and that sociodemographic disadvantages significantly increased the likelihood of substance use (9). In the presence of comorbidity, diagnostic assessment, treatment planning, adherence, and prognosis become more challenging; moreover, increased risks of suicide, violent behavior, higher readmission rates, and greater utilization of health care services are observed (10, 11). Therefore, investigating the prevalence of substance use disorders (SUDs), their clinical determinants, and their impact on treatment approaches among psychiatric inpatients appears essential both for improving individual clinical outcomes and for developing more effective interventions at the health system level.

Involuntary (compulsory) hospitalization refers to the admission and continuation of treatment of an individual without their consent. In Türkiye, this practice is regulated under Article 432 of the Turkish Civil Code (12). International studies have demonstrated that substance use disorders (SUDs) increase the likelihood of involuntary admission, with the risk being particularly elevated when co-occurring with psychotic disorders (13, 14). A limited number of studies from Türkiye have reported that substance use accounts for a substantial proportion of involuntary admissions (15). However, these studies have largely been limited to short-term observations; to date, no long-term research in Türkiye has examined temporal trends in the prevalence of substance use disorders (SUDs) among psychiatric inpatients, their relationship with involuntary admissions, and their implications for pharmacological treatment. Although numerous studies in the literature have investigated the prevalence and impact of SUDs within specific diagnostic groups such as bipolar disorder or schizophrenia, comprehensive investigations addressing SUD prevalence, substance use patterns, and their association with involuntary admissions across all psychiatric inpatients remain scarce. To our knowledge, no large-scale study of this scope has yet been conducted in Türkiye.

In this context, the present study aimed to retrospectively examine psychiatric inpatients admitted to psychiatry service between 2019 and 2023. Specifically, we sought to investigate (1) the prevalence and temporal trends of substance use disorders (SUDs) over a five-year period, (2) the trends in involuntary hospitalizations and the sociodemographic and clinical factors predicting involuntary admission, and (3) the impact of substance use and involuntary hospitalization on pharmacological treatment practices and their temporal trends. We anticipate that these findings will contribute to facilitating patient management in psychiatric clinics, providing a scientific basis for involuntary admission decisions, and informing the development of future mental health policies.

## 2 Methods

### 2.1 Study design and data collection

This study employed a retrospective and cross-sectional design. Between January 2019 and January 2024, the medical records of all patients hospitalized in the general psychiatric inpatient units, excluding the Alcohol and Substance Dependence Treatment Center of the University of Health Sciences Bursa Yuksek Ihtisas Training and Research Hospital were comprehensively reviewed. The total bed capacity of these wards is 120.

The hospital in which the study was conducted functions as a regional referral center providing mental health services to a wide geographical area. It receives a high volume of patient transfers from neighboring provinces, and the city in which it is located is also characterized by substantial migration from various regions of Türkiye.

During the study period, a total of 5,887 psychiatric inpatient admissions were identified: 1,469 in 2019, 1,035 in 2020, 1,072 in 2021, 1,303 in 2022, and 1,008 in 2023. The collected data included patients’ age, sex, substance use status, psychiatric diagnoses, and prescribed psychiatric treatments.

Each admission was recorded as a separate case, even in patients with multiple hospitalizations during the study period. This approach was chosen because restricting the analysis to index admissions could obscure clinically relevant information, such as the emergence of new comorbid substance use disorders or involuntary/legal admissions during subsequent hospitalizations. For each admission, the psychiatric diagnosis that prompted hospitalization was recorded, and therefore no exclusion was applied with regard to multiple or recurrent hospitalizations. Data from all admissions of patients aged 18 years and older were included in the analysis.

### 2.2 Statistical analysis

Descriptive statistics were presented as frequencies, percentages, means, and standard deviations. Differences in age and mean length of stay across years were assessed using one-way analysis of variance (ANOVA). Pearson’s chi-square test was applied to compare proportional variables between years. To identify factors predicting involuntary admission, binary logistic regression analysis was performed under the framework of generalized linear models (GLM) with a logit link function and binomial distribution assumption. Model fit was evaluated using McFadden’s pseudo-R², while classification performance was assessed by the area under the ROC curve (AUC), confusion matrix, and accuracy.

To examine temporal trends in psychiatric treatment modalities, simple linear regression analyses were conducted for each treatment type with year (time) as the independent variable. Slopes and coefficients of determination (R²) were interpreted to evaluate annual increases or decreases. In addition, to simultaneously model various psychiatric treatment types (e.g., SSRIs, electroconvulsive therapy, depot antipsychotics), seemingly unrelated regression (SUR) analysis was applied. SUR was preferred because the outcomes represent multiple treatment decisions made within the same clinical setting and patient population, and the unobserved factors affecting these treatment outcomes are likely correlated across equations. By accounting for cross-equation error correlation, SUR provides more efficient estimates and more reliable standard errors than fitting each equation separately when such correlations are present. This analysis was performed in Python 3.13 (64-bit) using the *linear models* package, and estimates were obtained with generalized least squares (GLS) employing heteroskedasticity-robust standard errors. In each regression equation, a different treatment type served as the dependent variable, while substance use, involuntary admission, age, sex, and year were included as independent variables.

Statistical analyses were conducted using both Python 3.13 (with the *stats models* and *linear models* packages) and IBM SPSS Statistics 26.0 (IBM Corp., Armonk, NY, USA). Normality was checked using skewness and kurtosis values (± 1.5), and statistical significance was set at p < 0.05 for all analyses.

## 3 Results

A total of 5887 patients were included in the study. The mean age of the participants was 37.5 ± 12.8 years, of whom 3858 (65.6%) were male and 2029 (34.4%) were female. The mean length of hospitalization was 25.2 ± 15.8 days. Adjudicated admissions were identified in 1656 patients (28.1%). The top five diagnoses were as follows: schizophrenia (SCH) with 2,477 patients (42.1%), bipolar affective disorder (BAB) with 1,335 patients (22.7%), depressive episode with 696 patients (11.8%), substance-induced psychosis (MBP) with 616 patients (10.5%), and substance use disorder with 553 patients (9.4%). When hospitalizations were examined across the study years, statistically significant differences were observed in substance use (X²=87.469, p<0.001), typical antipsychotic use (X²=35.313, p<0.001), atypical antipsychotic use (X²=236.063, p<0.001), clozapine use (X²=36.899, p<0.001), mood stabilizer (MS) (X²=24.203, p<0.001), and depot antipsychotic use (X²=13.620, p=0.009). In addition, the mean length of hospitalization (F = 25.084, p<0.001) differed significantly between years. The number of hospitalizations and the demographic and clinical characteristics of the patients across the study years are given in **Table 1**.

**Table 1:** The number of hospitalizations and the demographic and clinical characteristics of the patients across the study years.

Demographic and clinical characteristics		2019	2020	2021	2022	2023	p
Age, Mean ± SD		37.95 ± 12.81	36.22 ± 12.40	37.45 ± 13.13	37.36 ± 12.77	37.60 ± 12.62	0.018^§^
Length of Hospital Stay, Mean ± SD		22.59 ± 12.94	25.77 ± 17.02	25.22 ± 15.10	24.46 ± 16.92	28.91 ± 17.08	<0.001^§^
Gender, n (%)	Male	956 (65.1)	742 (71.7)	663 (61.8)	817 (62.7)	680 (67.5)	<0.001^†^
	Female	513 (34.9)	293 (28.3)	409 (38.2)	486 (37.3)	328 (32.5)	
Involuntary admission, n (%)	Yes	225 (15.3)	218 (21.1)	328 (30.6)	520 (39.9)	365 (36.2)	<0.001^†^
	No	1244 (84.7)	817 (78.9)	744 (69.4)	783 (60.1)	643 (63.7)	
Diagnosis, n (%)	BAD	344 (23.4)	224 (21.6)	214 (20.0)	295 (22.6)	258 (25.6)	<0.001^†^
	Psychotic Disorders	681 (46.4)	474 (45.8)	396 (36.9)	518 (39.8)	408 (40.5)	
	SUD	98 (6.7)	82 (7.9)	140 (13.1)	154 (11.8)	79 (7.8)	
	Substance-Induced Psychosis	88 (6.0)	113 (10.9)	137 (12.8)	170 (13.0)	108 (10.7)	
	Depressive Disorder	207 (14.1)	114 (11.0)	158 (14.7)	114 (8.7)	103 (10.2)	
	Diğer	51 (3.5)	28 (2.7)	27 (2.6)	52 (4.0)	52 (5.2)	
Substance Use, n (%)	Yes	271 (18.4)	291 (28.1)	344 (32.1)	419 (32.2)	294 (29.2)	<0.001^†^
	No	1198 (81.6)	744 (71.9)	728 (67.9)	884 (67.8)	714 (70.8)	
Typical Antipsychotic Use, n (%)	Yes	345 (23.5)	250 (24.2)	184 (17.2)	225 (17.3)	235 (23.3)	<0.001^†^
	No	1124 (76.5)	785 (75.8)	888 (82.8)	1078 (82.7)	773 (76.6)	
Atypical Antipsychotic Use, n (%)	Yes	885 (60.2)	706 (68.2)	819 (76.4)	1103 (84.7)	775 (76.9)	<0.001^†^
	No	584 (39.8)	329 (31.8)	253 (23.6)	200 (15.3)	233 (23.1)	
Clozapine, n (%)	Yes	99 (6.7)	45 (4.3)	57 (5.3)	94 (7.2)	107 (10.6)	<0.001^†^
	No	1370 (93.3)	990 (95.7)	1015 (94.7)	1209 (92.8)	901 (89.4)	
Mood stabilizer, n (%)	Yes	464 (31.6)	317 (30.6)	328 (30.6)	429 (32.9)	395 (39.2)	<0.001^†^
	No	1005 (68.4)	718 (69.4)	744 (69.4)	874 (67.1)	613 (60.8)	
Paliperidone palmitate depot (1-month), n (%)	Yes	330 (22.5)	293 (28.3)	272 (25.4)	378 (29.0)	296 (29.4)	<0.001^†^
	No	1139 (77.5)	742 (71.7)	800 (74.6)	925 (71.0)	712 (70.6)	
Zuclopenthixol depot, n (%)	Yes	79 (5.4)	31 (3.0)	31 (2.9)	57 (4.4)	42 (4.2)	0.009^†^
	No	1390 (94.6)	1004 (97.0)	1041 (97.1)	1246 (95.6)	966 (95.8)	
Aripiprazole depot, n (%)	Yes	78 (5.3)	91 (8.8)	145 (13.5)	150 (11.5)	94 (9.3)	<0.001^†^
	No	1391 (94.7)	944 (91.2)	927 (86.5)	1153 (88.5)	914 (90.7)	
Risperidone, n (%)	Yes	28 (1.9)	7 (0.7)	6 (0.6)	1 (0.1)	4 (0.4)	<0.001^†^
	No	1441 (98.1)	1028 (99.3)	1066 (99.4)	1302 (99.9)	1004 (99.6)	
Haloperidol Decanoate, n (%)	Yes	2 (0.1)	0 (0.00)	2 (0.2)	0 (0.0)	5 (0.5)	0.022^†^
	No	1467 (99.9)	1035 (100.0)	1070 (99.8)	1303 (100.0)	1003 (99.5)	
SSRI, n (%)	Yes	233 (15.9)	146 (14.1)	136 (12.7)	179 (13.7)	137 (13.6)	0.208^†^
	No	1236 (84.1)	889 (85.9)	936 (87.3)	1124 (86.3)	871 (86.4)	
SNRI, n (%)	Yes	123 (8.4)	59 (5.7)	71 (6.6)	115 (8.8)	80 (7.9)	0.027^†^
	No	1346 (91.6)	976 (94.3)	1001 (93.4)	1188 (91.2)	928 (92.1)	
Atypical Antidepressant, n (%)	Yes	86 (5.9)	51 (4.9)	60 (5.6)	75 (5.8)	33 (3.3)	0.037^†^
	No	1383 (94.1)	984 (95.1)	1012 (94.4)	1228 (94.2)	975 (96.7)	
ECT, n (%)	Yes	159 (10.8)	78 (7.5)	86 (8.0)	140 (10.7)	182 (18.1)	<0.001^†^
	No	1310 (89.2)	957 (92.5)	986 (92.0)	1163 (89.3)	826 (81.9)	

†=Pearson Chi-square test, §=One-way ANOVA test. BAD, Bipolar Affective Disorder; SUD, Substance Use Disorder; SSRI, Selective Serotonin Reuptake Inhibitor; SNRI, Serotonin-Norepinephrine Reuptake Inhibitor; ECT, Electroconvulsive Therapy.

**Figure 1:**
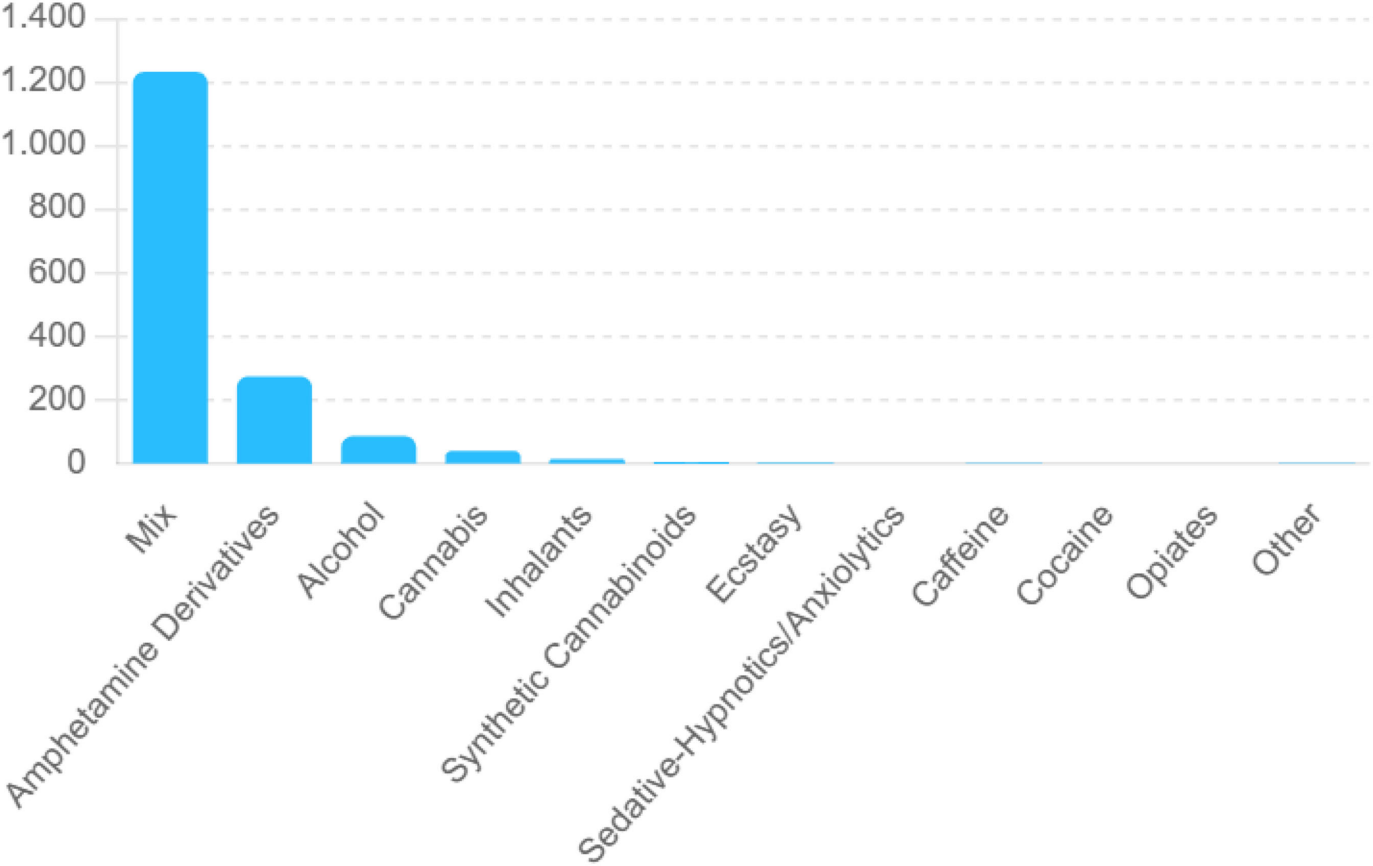
Distribution of substance use patterns among psychiatric inpatients (2019–2023).

Between 2019 and 2023, a total of 5,887 patient admissions were evaluated. Among these patients, substance use was identified in 1,619 cases (27.5%), while 4,268 patients (72.5%) had no history of substance use. Among those who used substances, the most common pattern was mixed substance use (Mix), observed in 1,232 cases (76.1%). This was followed by amphetamine derivatives with 274 cases (16.9%) and alcohol use with 87 cases (5.4%). Lower rates were observed for cannabis with 40 cases (2.5%), inhalants with 14 cases (0.9%), synthetic cannabinoids with 5 cases (0.3%), and ecstasy with 2 cases (0.1%) (**Figure 1**).

To identify the factors predicting involuntary hospitalization, a binary logistic regression analysis was conducted within the framework of a Generalized Linear Model (GLM). A logit (logistic) link function was applied (McFadden’s Pseudo R² = 0.1283, area under the ROC curve [AUC] = 0.7384). In the regression analysis, the diagnostic group “psychosis and related disorders” was used as the reference category. This reference category comprised schizophrenia spectrum and other primary psychotic disorders classified under ICD-10 codes F20–F29, excluding substance-induced psychotic disorders. Compared to this group, individuals diagnosed with bipolar affective disorder (BAD) had a significantly lower risk of involuntary admission (OR = 0.3885, 95% CI: 0.3223–0.4681, p < 0.001). In contrast, patients with substance use disorder (SUD) (OR = 3.6305, 95% CI: 2.9652–4.4451, p < 0.001) and substance-induced psychotic disorder (OR = 2.3270, 95% CI: 1.9237–2.8148, p < 0.001) showed a significantly higher risk of involuntary hospitalization. The continuous variable “time (year)” also exerted a positive and significant effect (OR = 1.4021, 95% CI: 1.3411–1.4659, p < 0.001), indicating that the likelihood of involuntary admissions increased over time. While age was not a significant predictor (p = 0.2614), male sex was associated with a higher risk compared to females (OR = 1.3009, 95% CI: 1.1253–1.5040, p < 0.001). The results of the regression analysis identifying predictors of involuntary hospitalization are presented in **Table 2**.

**Table 2:** Regression analysis of factors predicting involuntary hospitalization.

Variable	B	SE	p	OR	95% CI	
					LL	UL
Intercept	-684.6989	45.8770	<0.001	0.0000	0.0000	0.0000
BAD	-0.9456	0.0952	<0.001	0.3885	0.3223	0.4681
SUD	1.2894	0.1033	<0.001	3.6305	2.9652	4.4451
Substance-Induced Psychosis	0.8446	0.0971	<0.001	2.3270	1.9237	2.8148
Other diagnoses	-0.7612	0.1069	<0.001	0.4671	0.3788	0.5760
Year (Time)	0.3380	0.0227	<0.001	1.4021	1.3411	1.4659
Age	-0.0031	0.0028	0.2614	0.9969	0.9916	1.0023
Gender	0.2631	0.0740	<0.001	1.3009	1.1253	1.5040

A binary logistic regression analysis was conducted under the framework of a Generalized Linear Model (GLM), using a logit link function as the link function. McFadden Pseudo R² = 0.1283, AUC = 0.7384, accuracy = 75.76%.

For the gender variable, the reference group is female; for the diagnosis variable, the reference category is “psychosis and related disorders” (ICD-10 F20–F29, excluding substance-induced psychotic disorders). BAD, Bipolar Affective Disorder; SUD, Substance Use Disorder.

**Figure 2:**
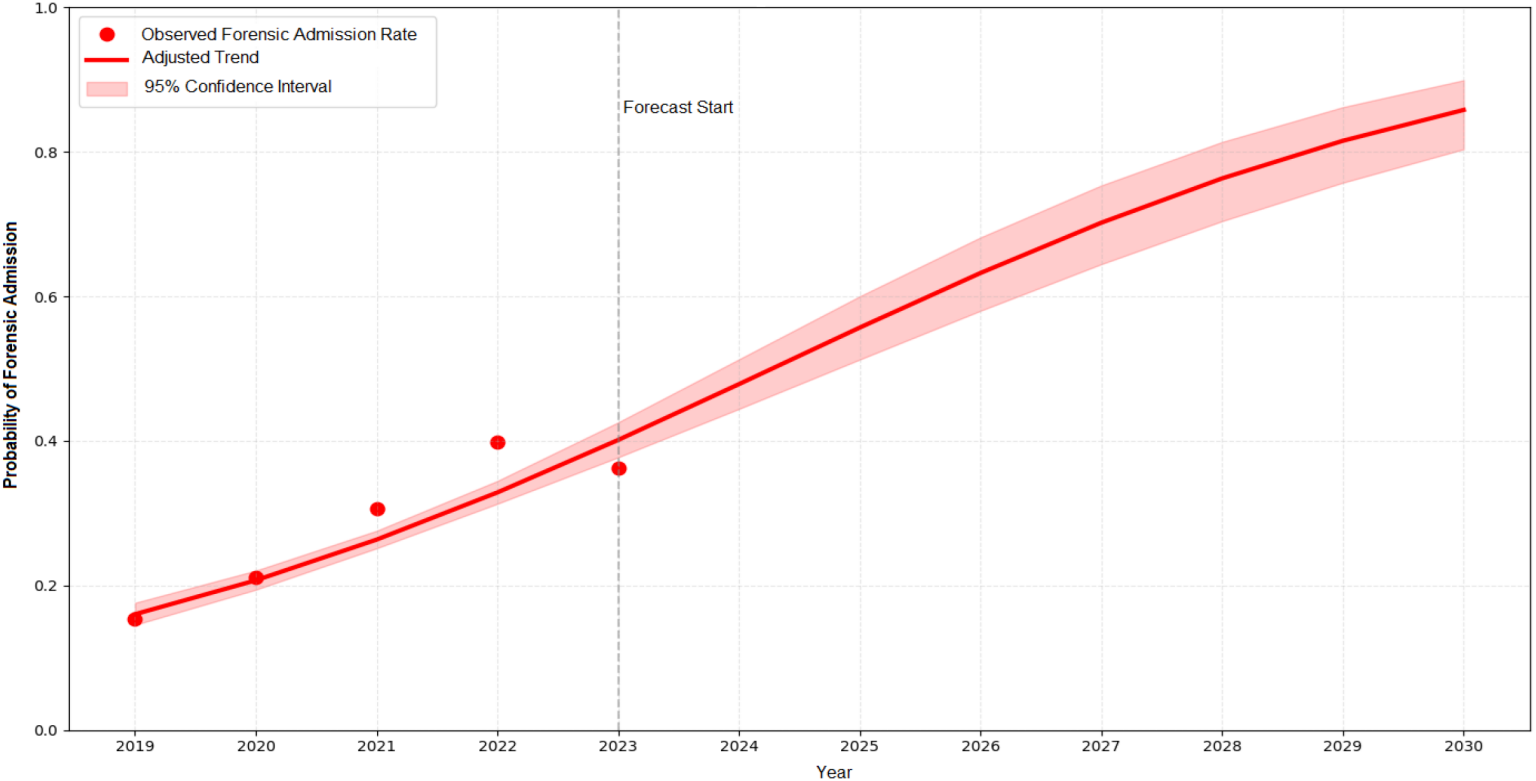
Trend analysis of involuntary hospitalization probability by year. The actual involuntary hospitalization rates (red dots), the trend predicted by the logistic regression model adjusted for age, gender, and substance use (red line), and the 95% confidence interval (red shaded area) are shown for the period 2019–2030. The year 2023 marks the beginning of the model’s forecast period (gray dashed line).

The logistic regression analysis revealed that time (β = 0.314, p < 0.001), sex (male; β = 0.343, p < 0.001), and substance use (β = 1.206, p < 0.001) exerted significant and positive effects on the likelihood of involuntary hospitalization. In contrast, age was not a statistically significant predictor in the model (β = –0.004, p = 0.134). Based on the Pseudo R² value (CS = 0.1151), the overall model fit was considered acceptable. **Figure 2** presents the observed rates of involuntary hospitalization (2019–2023) alongside the adjusted trend estimated by the model with 95% confidence intervals. These results indicate a steady increase in the probability of involuntary admissions over time, with higher risks observed among male patients and those with substance use.

**Figure 3:**
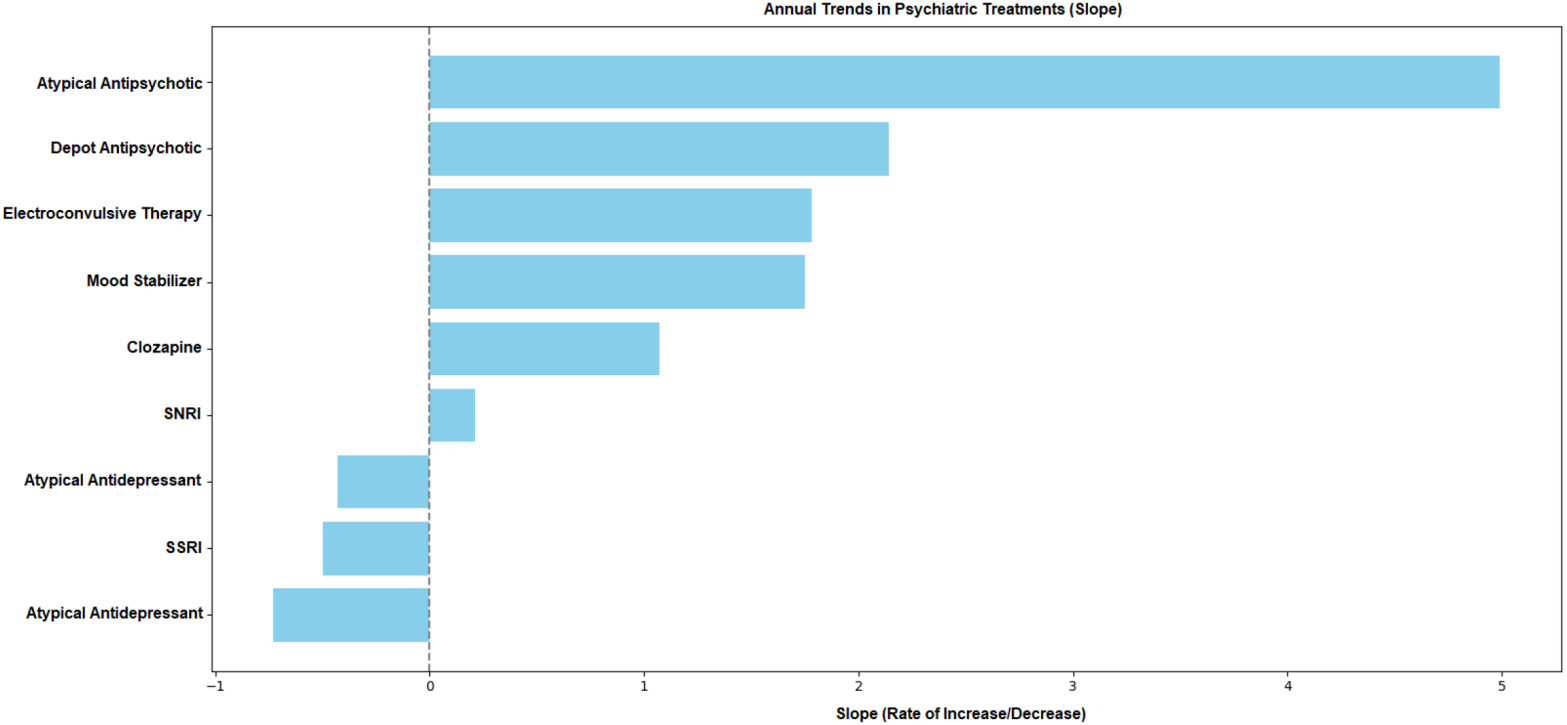
Trends in psychiatric treatment by year. Rates of change (slope values) in psychiatric treatment trends by year. Positive slope values indicate an increase in the use of treatment methods over the years, while negative slopes indicate a decrease.

In the study, trends in psychiatric treatment approaches between 2019 and 2023 were evaluated using linear regression analysis. For each treatment type, year was entered as the independent variable, while the annual rate of use was defined as the dependent variable. Accordingly, the regression slope, intercept, and coefficient of determination (R²) were calculated for each treatment modality. Examination of treatment trends over the five-year period revealed the greatest increase in atypical antipsychotics, followed by depot antipsychotics, electroconvulsive therapy (ECT), mood stabilizers (MS), and clozapine. A slight upward trend was observed in the SNRI group, whereas the use of atypical antidepressants, SSRIs, and typical antipsychotics showed a declining trend (**Figure 3**).

According to the results of the Seemingly Unrelated Regression (SUR) analysis, the variable “year” was found to significantly increase the use of atypical antipsychotics (β = 0.0533, p <.001), clozapine (β = 0.0117, p <.001), MS (β = 0.0252, p <.001), electroconvulsive therapy (ECT) (β = 0.0192, p <.001), and depot antipsychotics (β = 0.0218, p <.001). Substance use was associated with a decrease in the use of typical antipsychotics (β = –0.0447, p <.01), clozapine (β = –0.0399, p <.001), MS (β = –0.1236, p <.001), and ECT (β = –0.0578, p <.001), while increasing the use of atypical antipsychotics (β = 0.0567, p <.001) and atypical antidepressants (β = 0.0178, p <.01). Involuntary hospitalization was found to increase the use of depot antipsychotics (β = 0.0470, p <.01), but to reduce the use of clozapine, MS, SNRIs, and ECT (**Table 3**).

**Table 3:** Effects of year, substance use, and involuntary hospitalization on types of psychiatric treatment (SUR estimates).

Variable	Typical antipsychotic	Atypical antipsychotic	Clozapine	Mood stabilizer	SSRI	SNRI	Atypical antidepressant	Depot	ECT
Year (time)	-0.0064 (0.0039)	0.0533 (0.0041) ***	0.0117 (0.0026) ***	0.0252 (0.0043) ***	-0.0037 (0.0033)	0.0028 (0.0025)	-0.0036 (0.0020)	0.0218 (0.0045) ***	0.0192 (0.0031) ***
Substance Use	-0.0447 (0.0132) **	0.0567 (0.0144) ***	-0.0399 (0.0087) ***	-0.1236 (0.0144) ***	-0.0210 (0.0108)	0.0145 (0.0074)	0.0178 (0.0065) **	-0.0381 (0.0164) *	-0.0578 (0.0098) ***
Involuntary Hospitalization	-0.0173 (0.0124)	-0.0229 (0.0135)	-0.0226 (0.0078) **	-0.1033 (0.0134) ***	-0.0189 (0.0101)	-0.0271 (0.0069) ***	-0.0156 (0.0060) **	0.0470 (0.0153) **	-0.0408 (0.0088) ***

Unstandardized beta coefficients are presented with robust standard errors in parentheses. p <.05, ** p <.01, *** p <.001. Estimates are based on Seemingly Unrelated Regression (SUR) analysis conducted using the Generalized Least Squares (GLS) method with robust standard errors. The results are adjusted for age and gender. Coefficients reflect the effects of year, substance use, and involuntary hospitalization on each dependent variable. SSRI: Selective Serotonin Reuptake Inhibitor, SNRI, Serotonin-Norepinephrine Reuptake Inhibitor; ECT, Electroconvulsive Therapy.

## 4 Discussion

In this study, 5,887 psychiatric inpatient admissions between 2019 and 2023 were evaluated to examine the prevalence of substance use disorders (SUDs), their association with involuntary hospitalization, and their impact on treatment approaches. Our findings indicate that SUDs were significantly associated with involuntary admission and with differences in pharmacological treatment choices. Furthermore, the probability of compulsory hospitalization was found to increase over the years, with a higher risk observed among male patients. In terms of treatment trends, there was a marked increase in the use of atypical antipsychotics, depot antipsychotics, clozapine, and electroconvulsive therapy (ECT).

One of the most striking findings of our study is that SUD was strongly associated with involuntary hospitalization. This result is consistent with previously reported findings in the literature. Schmidt et al. similarly reported that comorbid SUD in patients with schizophrenia was linked to higher rates of involuntary admissions and poorer long-term outcomes (8). Similarly, other studies have reported that the risk of involuntary hospitalization is markedly increased among individuals with substance use, particularly when accompanied by psychotic disorders (13, 14). Our study strengthens this association by providing long-term data from a large sample of psychiatric inpatients in Türkiye.

The finding that males had a higher risk of involuntary hospitalization is consistent with gender differences reported both in Türkiye and in the international literature. A multicenter study conducted in Norway reported that male sex increased the likelihood of involuntary admission (16). Similarly, a study from Brazil comparing voluntary and involuntary hospitalizations found that men were significantly overrepresented among involuntarily admitted patients (17). Several factors may contribute to this pattern, including differences in clinical presentation at admission (e.g., agitation or behavioral dysregulation), variations in social support, and differences in help-seeking behavior.

In our study, 9.4% of patients were diagnosed with substance use disorder (SUD). Notably, even in the absence of a comorbid primary psychiatric diagnosis, these individuals often exhibited self-harming or harmful behaviors toward others related to substance use and were admitted involuntarily, frequently under family pressure. Moreover, the proportion of such cases showed an increasing trend over the study years. Although Türkiye has specialized treatment centers for individuals with SUD (AMATEM), a substantial number of these patients continue to be involuntarily admitted to general psychiatric wards under Article 432 of the Turkish Civil Code. In many cases, this practice occurs as a result of family insistence and the compulsory decision-making process of clinicians, raising ongoing debates regarding both patient rights and clinical management. However, by its very nature, SUD is considered a condition more effectively addressed through voluntary and motivational approaches (18). Involuntary treatment practices in substance use disorders are not unique to our country; they have long been implemented in other countries as well and remain a highly controversial issue (14, 19). The main reasons for resorting to involuntary treatment include patients’ refusal to accept treatment voluntarily, the high risk of harm to themselves or others while under the influence of substances, poor treatment adherence, and the need to ensure public safety (19, 20). However, there is no clear consensus in the literature regarding the effectiveness of such practices. Indeed, a comprehensive systematic review published by Bahji et al. in 2023, which included 42 studies, reported that involuntary treatments predominantly increased the length of stay in treatment but did not demonstrate significant or lasting benefits in reducing substance use or improving functioning (20). In general psychiatric wards, patients who are involuntarily admitted for substance use disorder are likely to show poor treatment adherence, limited treatment effectiveness, and to impose additional pressure on clinicians. Our findings indicate that the management of SUD through involuntary hospitalization is becoming an increasingly important issue in Türkiye and highlight the need for clearer legal, ethical, and clinical regulations in this area.

In our study, diagnostic distribution revealed that psychotic disorders (40.8%), bipolar disorders (22.7%), and substance use disorders (9.4%) were the most common, together accounting for more than two-thirds of all patients. This pattern suggests that the study center may primarily serve acute cases with a high likelihood of involuntary admission. Similarly, in Türkiye, mental health services appear to be increasingly concentrated on severe psychotic episodes, manic episodes, and substance use–related cases requiring compulsory hospitalization. By contrast, a nationwide study conducted in Brazil reported a marked decrease in hospitalizations related to schizophrenia and associated disorders, attributed to the strengthening of community-based mental health policies (21). In another study conducted in New Zealand by Chai et al., it was found that a substantial proportion (64%) of psychiatric hospitalizations consisted of patients diagnosed with depressive disorders, anxiety disorders, and personality disorders, while those with bipolar affective disorder accounted for less than 10% (22). In these countries, the relatively more advanced development of community-based mental health services allows a broader range of patient groups to benefit from inpatient care. This is considered an important reason for the differences in diagnosis–hospitalization ratios compared to Türkiye. Furthermore, the implementation of preventive measures, social integration programs, and strengthened community-based policies for chronic mental disorders has been identified as a key factor in reducing the hospital burden of severe psychotic cases. Taken together, these differences highlight the need to further develop community-based services in Türkiye.

In terms of psychiatric treatment trends, our study found a marked increase in the use of atypical antipsychotics and a decline in the use of typical antipsychotics. Atypical antipsychotics are widely used in the treatment of bipolar disorder, psychotic disorders, depressive disorders, and substance use disorders. Moreover, the less favorable side effect profiles of typical antipsychotics, including extrapyramidal symptoms and tardive dyskinesia, have led to their decreasing preference in clinical practice. In this respect, our findings appear to be consistent with the recommendations of international treatment guidelines and general clinical practice (23–25). Additionally, our study demonstrated an increasing use of depot antipsychotics, a finding consistent with the literature emphasizing their role in enhancing adherence and reducing the risk of relapse and rehospitalization (26).

When the impact of substance use on treatment approaches was evaluated, a decrease was observed in the use of typical antipsychotics, clozapine, MS, and electroconvulsive therapy (ECT), whereas the use of atypical antipsychotics and atypical antidepressants increased. In a review published by Zhornitsky et al. in 2010, it was reported that atypical antipsychotics were more effective than typical antipsychotics in reducing alcohol and cannabis use among individuals with comorbid substance use disorder and psychosis. In contrast, typical antipsychotics were found to be not only less effective but also associated with an increased risk of relapse in some cases (27). These findings may reflect clinicians’ efforts to balance efficacy, tolerability, and behavioral symptom management in patients with comorbid substance use (7, 28). Atypical antipsychotics are particularly preferred in patients with comorbid substance use disorders due to their more tolerable side-effect profile and their effectiveness on behavioral symptoms. Similarly, increased use of antidepressants has been reported in this group, as depressive symptoms and anxiety are highly prevalent among these patients (29, 30).

This study has several limitations that should be acknowledged. First, its retrospective and observational design relied on medical records, making the accuracy of the data dependent on the completeness and quality of documentation; therefore, although associations between the findings were observed, no causal inferences can be drawn. Second, each hospitalization was treated as a separate observation, including repeated admissions of the same individual. This approach may have violated the assumption of independence, as within-patient correlations could not be accounted for, potentially leading to biased standard errors. Third, as a single-center study conducted in a regional referral hospital, the findings may not be fully generalizable to other psychiatric settings or to the national level. Fourth, the variables available for analysis were limited, as potentially influential factors such as socioeconomic status, family history, social support, employment status, and treatment adherence could not be assessed. Finally, although the study period includes the COVID-19 pandemic years (2020–2022), no separate sensitivity or stratified analyses were conducted to isolate pandemic-related effects. Therefore, changes in hospitalization patterns and substance use behaviors during this period may have partially influenced the observed trends.

In this large-scale retrospective study of psychiatric inpatients, substance use disorders were found to be highly prevalent and strongly associated with an increased risk of involuntary hospitalization as well as significant differences in pharmacological treatment practices. Over the five-year study period, the likelihood of compulsory admissions increased, particularly among male patients and those with substance-related diagnoses. The findings emphasize the urgent need for more structured approaches to the management of comorbid substance use in psychiatric settings, including the development of evidence-based guidelines, ethical and legal frameworks for involuntary admissions, and the expansion of community-based services to reduce reliance on compulsory hospitalization. Addressing these issues is crucial not only for improving patient outcomes but also for informing future mental health policies in Türkiye.

## Data Availability

The dataset contains sensitive and confidential patient information, including psychiatric diagnoses, demographic details, and clinical treatment data. Access to this dataset is restricted to protect patient privacy and comply with national data protection laws (Turkish Personal Data Protection Law - KVKK) and the requirements of the institutional ethics committee. The data are therefore not publicly available and cannot be included in the article or supplementary materials. However, anonymized data that support the findings of this study can be made available from the corresponding author upon reasonable request, subject to a data sharing agreement and approval from the original approving ethics committee. Requests to access these datasets should be directed to EA, emervekalyoncu@gmail.com.
